# Case report of periorbital metastasis from rectal cancer

**DOI:** 10.1097/MD.0000000000018479

**Published:** 2020-01-03

**Authors:** Jin-Hee Paik, Hyun Jin Shin, Hye Seung Lee, Hye Seung Han, Chun-Geun Ryu, Dae-Yong Hwang

**Affiliations:** a Department of Surgery, Colorectal Cancer Center; b Department of Ophtalmology, Medicine; c Department of Pathology; d Department of Surgery, Konkuk University Medical Center, Konkuk University School of Medicine, Gwangjin-gu, Seoul, Republic of Korea.

**Keywords:** eyelid neoplasms, neoplasm metastasis, rectal neoplasms

## Abstract

**Introduction:**

Periorbital metastasis of colorectal cancer is rare. Therefore, herein, we report a patient with rectal cancer who presented with periorbital metastasis without any systemic metastasis.

**Patient concerns:**

The patient was a 57-year-old man who had a painless nodule on his left eyelid.

**Diagnosis:**

The patient presented with loose and frequent stools and was diagnosed with rectal adenocarcinoma via colonoscopic biopsy at the local clinic. Curative resection (low anterior resection with temporary ileostomy formation) was performed 4 weeks after completing chemoradiotherapy. The final TNM stage was yp stage T2N0M0. Eight months after the diagnosis of rectal cancer, a protruding lesion was noticed on the patient's left eyelid. Histologic evaluation of the nodule revealed metastatic adenocarcinoma of rectal cancer.

**Interventions:**

The patient received neoadjuvant chemoradiotherapy and curative resection for rectal cancer. After excision of the periorbital nodule, he received 5 cycles of chemotherapy.

**Outcomes:**

The patient underwent regular follow-up because he was not able to endure chemotherapy; no recurrence has been observed 21 months after the diagnosis of rectal cancer. Histologic examination revealed metastatic adenocarcinoma of rectal cancer on the patient's left eyelid. However, consecutive imaging studies revealed no other metastatic lesions. Finally, the patient was diagnosed with a solitary periorbital metastasis of rectal cancer.

**Conclusion:**

This case report helps in understanding the course of progression from rectal cancer to periorbital metastasis.

## 1 Introduction

Colorectal cancer (CRC) is the third most common cancer in men and second most common cancer in women worldwide.^[1]^ As early CRC does not present with typical symptoms, most cases of CRC are diagnosed in advanced stages. Approximately 25% of patients with CRC have distant metastasis at the time of diagnosis. Common metastatic sites of CRC include the liver, lungs, distant lymph nodes, and peritoneum.^[2,3]^ However, orbital metastasis of CRC is extremely rare. Only 4% of gastrointestinal tract cancers have been reported to metastasize to the ocular region.^[4]^

Herein, we report a rare case of periorbital metastasis in a patient who underwent neoadjuvant chemoradiotherapy and surgery for rectal cancer.

## 2 Case report

**Figure 1:**
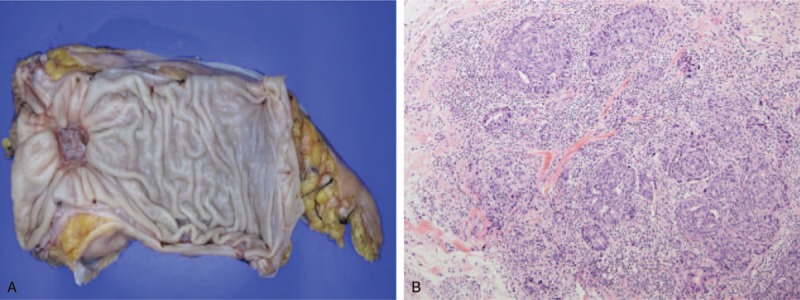
Gross pathology (A) and microscopic finding, (B) of rectal adenocarcinoma.

A 57-year-old man without a significant medical history visited the colorectal center of our hospital for further work-up and treatment of rectal cancer detected at a local clinic. He had presented with loose and frequent stools when he was first diagnosed with rectal adenocarcinoma via colonoscopic biopsy at the local clinic. Colonoscopic findings indicated a 4-cm encircling rectal mass without a movable anterior side. Pelvic magnetic resonance imaging revealed several enlarged regional lymph nodes. We planned to perform neoadjuvant chemoradiotherapy and curative resection assuming clinical stage III disease (T3N2M0). First, the patient underwent 23 cycles of preoperative radiotherapy, consisting of whole pelvic radiation at a dose of 46 Gy and boost radiation of 2 Gy. Chemotherapy was then administered with 3 cycles of 5-fluorouracil (425 mg/m^2^) and leucovorin (20 mg/m^2^) every 4 weeks. Curative resection (low anterior resection with temporary ileostomy formation) was performed 4 weeks after chemoradiotherapy was completed. The pathology report indicated a moderately differentiated adenocarcinoma with yp stage T2N0M0 disease, according to the TNM stage (Fig. 1). Ileostomy repair was performed 3 months later, and postoperative care was initiated.

**Figure 2:**
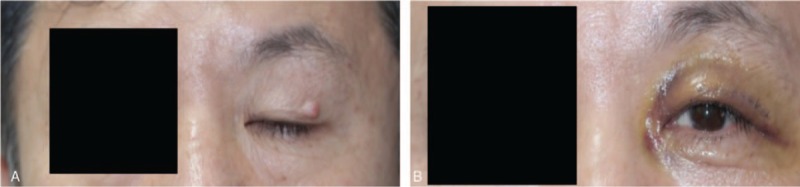
Gross image of the left eye of the patient (A: preoperative, B: postoperative).

**Figure 3:**
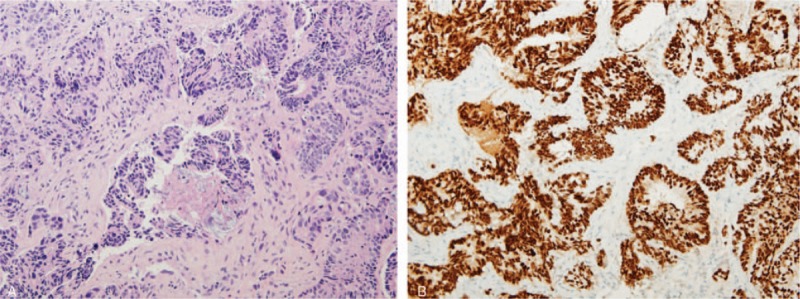
Pathologic findings of periorbital nodule. (A) The slide shows typical microscopic appearance of adenocarcinoma. Centeral necrosis can explain as a metastasis from rectal cancer rather than an adenocarcinoma from periorbital mucosa. (B) The slide shows Cytokeratin 7 negative, Cytokeratin 20 positive, Caudal type homeobox 2 positive that means carcinoma from lower gastrointestinal tract.

Eight months after the diagnosis of rectal cancer, a nodule was noticed on the patient's left eyelid (Fig. 2A, B). The protruding lesion on his left eyelid was noticed at the time of ileostomy repair. The patient was then referred to the ophthalmology department where en-bloc resection was performed for the eyelid lesion, including the skin, subcutaneous tissue, and muscle. Histologic evaluation of the specimen revealed metastatic adenocarcinoma of rectal cancer on the eyelid (Fig. 3), with a clear resection margin (upper margin, 1.0 mm; lower margin, 0.5 mm; lateral margin, 10.0 mm; surgical margin, 2.0 mm; and deep margin, 0.1 mm). Neither brain magnetic resonance imaging nor neck computed tomography revealed evidence of intracranial or cervical metastasis. Total whole body fluorodeoxyglucose-fusion positron emission tomography also revealed no significant abnormal hypermetabolic lesions that were indicative of malignancy.

The patient then received chemotherapy with the FOLFOX (fluorouracil plus leucovorin and oxaliplatin) regimen, but he was not able to tolerate the treatment after the fifth cycle. Therefore, he stopped receiving chemotherapy, and periodic follow-up examinations were performed. The patient showed no recurrence at the last follow-up, i.e., 13 months after excision for the nodule on the left eyelid.

## 3 Discussion

Survival after CRC diagnosis has been prolonged owing to the development of new chemotherapeutic modalities, including targeted agents. With the improvement in survival, CRC has an increased tendency to metastasize to rare sites, such as the bones, brain, and spleen. Nonetheless, periocular metastasis of CRC is extremely rare. Moreover, solitary periocular metastasis is uncommon among other malignancies. Furthermore, systemic metastasis of breast cancer is rare in the periorbital region.^[5]^ Distant systemic metastases are more common in rectal cancer than in colon cancer. This may be because of the venous circulatory system in rectal cancer, which allows rectal cancer metastases to better reach the periorbital region, compared to colon cancer.^[6]^

Hematogenous dissemination of colon cancer is predominantly through 1 tract in the portal venous circulation, whereas rectal cancer leads to systemic metastasis via 2 routes: pulmonary circulation to the ophthalmic artery and seeding on Batson venous plexus to the ophthalmic vein through the vertebral plexus.^[6,7]^ These metastatic routes of rectal cancer are likely to cause metastasis at other sites, including the lungs and the vertebral column. In a previous study, infiltrative brain metastasis was associated with periocular metastasis of rectal cancer. The patient died 7 months after initial chemotherapy because of metastasis to other organs, including the bone and pleura.^[8]^ However, in the current case, only a solitary periorbital metastasis was observed, without other metastatic lesions. Therefore, the current case may be an example of tumor embolism, which can be explained by the hypothesis used to explain systemic metastasis of rectal cancer.^[9]^

Patients with periorbital metastasis generally complain of symptoms such as painful nodules, diffuse eyelid swelling, diplopia, and blurred vision. In general, the prognosis of patients with orbital metastases is poor, with a median survival rate of approximately 10 to 20 months.^[10,11]^ In the present case, the patient did not have any specific symptoms, including visual disturbance or eyeball pain. Despite the absence of standard treatments for periorbital metastasis of rectal cancer, we administered additional chemotherapy for the treatment of systemic metastasis.

In conclusion, we reported a case of solitary periorbital metastasis of rectal cancer without other distant metastatic lesions. This case report helps in understanding the course of progression from rectal cancer to periorbital metastasis. Further studies should be conducted to establish the proper management for solitary periorbital metastasis of rectal cancer.

## Author contributions

**Data Curation:** Hyun Jin Shin.

**Methodology:** Hye Seung Lee, Hye Seung Han.

**Supervision:** Dae-Yong Hwang.

**Validation:** Chun Geun Ryu.

**Writing – original draft:** Jin-Hee Paik.

**Writing – review & editing:** Dae-Yong Hwang.

Dae-Yong Hwang orcid: 0000-0001-9082-8431.
